# Dynamic colonization history in a rediscovered Isle Royale carnivore

**DOI:** 10.1038/s41598-018-31130-0

**Published:** 2018-08-23

**Authors:** Philip J. Manlick, Mark C. Romanski, Jonathan N. Pauli

**Affiliations:** 1 University of Wisconsin – Madison, Department of Forest & Wildlife Ecology, Madison, WI, USA; 2 National Park Service, Isle Royale National Park, Houghton, Michigan, USA

## Abstract

Island ecosystems are globally threatened, and efforts to restore historical communities are widespread. Such conservation efforts should be informed by accurate assessments of historical community composition to establish appropriate restoration targets. Isle Royale National Park is one of the most researched island ecosystems in the world, yet little is actually known about the biogeographic history of most Isle Royale taxa. To address this uncertainty and inform restoration targets, we determined the phylogeographic history of American martens (*Martes americana*), a species rediscovered on Isle Royale 76 years after presumed extirpation. We characterized the genetic composition of martens throughout the Great Lakes region using nuclear and mitochondrial markers, identified the source of Isle Royale martens using genetic structure analyses, and used demographic bottleneck tests to evaluate (eliminate redundancy of test). 3 competing colonization scenarios. Martens exhibited significant structure regionally, including a distinct Isle Royale cluster, but mitochondrial sequences revealed no monophyletic clades or evolutionarily significant units. Rather, martens were historically extirpated and recolonized Isle Royale from neighbouring Ontario, Canada in the late 20^th^ century. These findings illustrate the underappreciated dynamics of island communities, underscore the importance of historical biogeography for establishing restoration baselines, and provide optimism for extirpated and declining Isle Royale vertebrates whose reintroductions have been widely debated.

## Introduction

Island ecosystems, and the unique taxa they feature, have fascinated biologists since the inception of ecology and evolution^1–3^. Due to their seeming simplicity, islands are often useful models to understand ecological interactions^4,5^ and the evolutionary histories of regional taxa^6^. Moreover, islands have served as refugia in the face of global change throughout history^7,8^, often resulting in endemic species or unique genetic lineages due to long-term isolation^9^. Yet, contemporarily, islands are some of the most altered ecosystems^10^ and are regularly subject to introduced species^11^, novel diseases^12^, and overexploitation^13^. Consequently, extensive conservation programs have been established to protect island biodiversity^14,15^ and to restore departed island communities^16,17^. Historical community composition, though, is not always clear, leading to ambiguous restoration baselines and the potential mismanagement of native and invasive species^18,19^.

Isle Royale National Park is an isolated archipelago in the western reaches of Lake Superior, USA. A national park since 1931 and a designated wilderness area since 1976, Isle Royale is widely regarded as one of the most pristine island ecosystems in the world^20,21^. Moreover, Isle Royale is home to the longest running predator-prey study on record, resulting in a comprehensive understanding of wolf (*Canis lupus*)-moose (*Alces alce*s) interactions, and a deeper appreciation for paired population and trophic dynamics^4,22,23^. Recent declines in wolf abundance have also sparked widespread discussions on conservation ethics, wilderness management, and the restoration of island communities^20,24,25^. Despite this attention, little is actually known about the 17 other mammals that inhabit Isle Royale, and widespread species turnover throughout the 20^th^ century has obscured historical community composition^26^. Indeed, historical assemblages of vertebrates have been constructed entirely from museum surveys and anecdotal accounts^27,28^. Furthermore, lake ice formation, a primary mode of colonization to Isle Royale, has become increasingly stochastic due to regional climatic warming, potentially disrupting historical connectivity to the mainland^29^. Thus, the restoration of Isle Royale fauna is confounded by uncertainties in both historical community composition and future colonization potential^26^. Nevertheless, the reintroduction of Isle Royale carnivores has garnered widespread consideration^30,31^ and illuminated the need for *a priori* restoration baselines derived from phylogenetic histories of past and present community members^26^.

Prior to establishment as a National Park, Isle Royale was subject to significant anthropogenic disturbances at the turn of the 20^th^ century that included the extirpation of Canada lynx (*Lynx canadensis*), woodland caribou (*Rangifer tarandus caribou*), and, presumably, American martens (*Martes americana*; hereafter martens)^32^. Though historically abundant on the island^27^, martens were valuable furbearers and trapped heavily until 1917 when the last recorded specimen was collected^33^. Following four decades of presumed absence, the National Park Service initiated a program to reintroduce martens from Ontario, Canada to Isle Royale in 1966; however, the translocation of martens to the island was never documented, though such a release cannot be completely discounted^34^. A quarter century later marten tracks were observed, and in 1993 martens were once again confirmed on Isle Royale^34^. Martens have since remained rare following this apparent 76-year absence, and the origins of this extant population are unknown.

**Figure 1:**
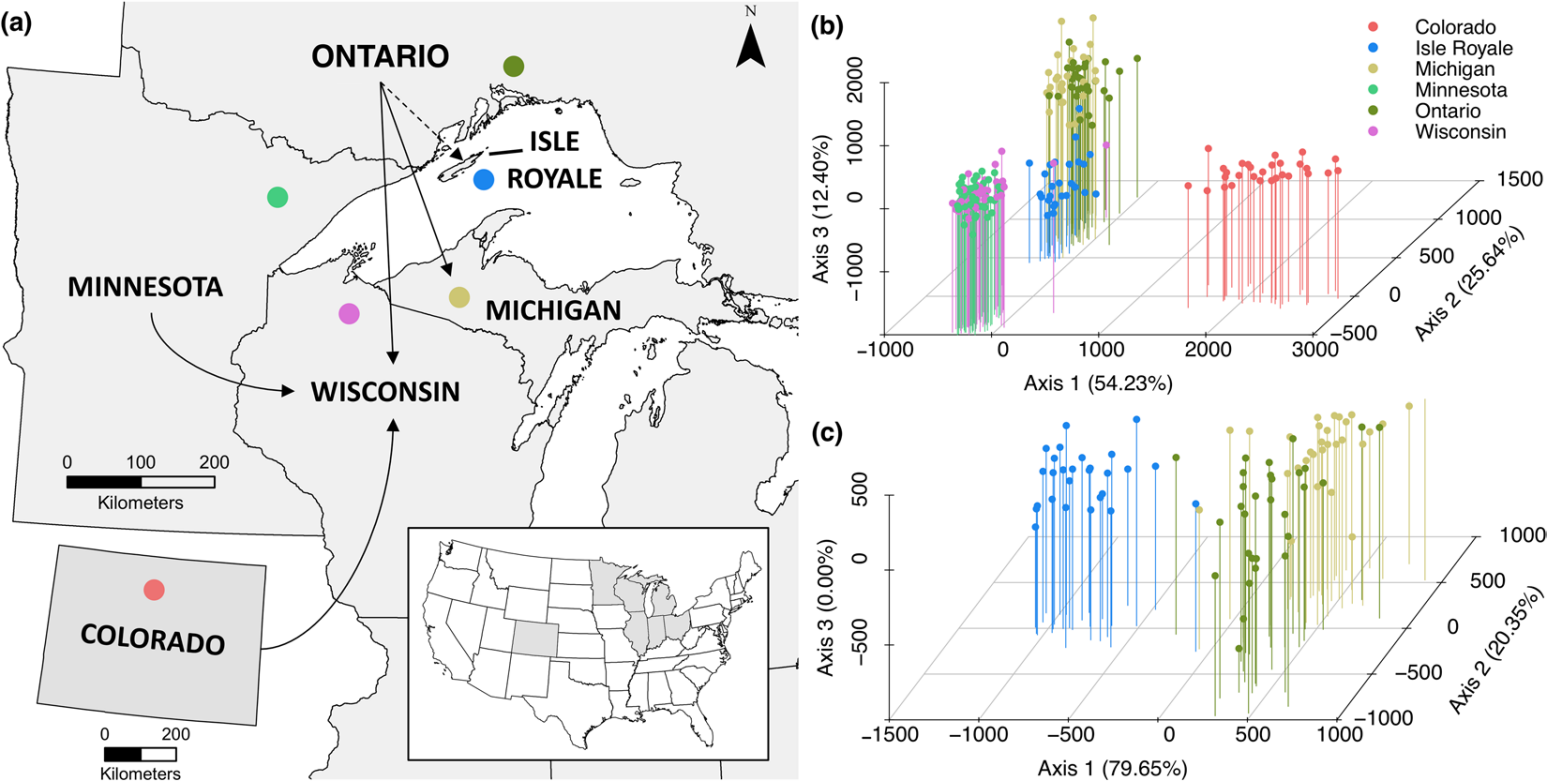
(**a**) Distribution of sampled sites in the Lake Superior basin, and the location of historical reintroductions (solid lines) as well as the potential reintroduction of martens to Isle Royale in 1966 (dashed line). Points and colours correspond to sampling locations and population clusters identified using factorial correspondence analysis (FCA) of microsatellite loci across all sites (**b**). Fine scale structure was detected, with Isle Royale segregating from Michigan and Ontario martens (**c**).

Isle Royale has been isolated *c*. 11,000 years, resulting in unique lineages of several taxa^35,36^. Like other historical community members (e.g. Isle Royale red squirrels [*Tamiasciurus hudsonicus regalis*]^36^), martens could constitute an evolutionarily unique population. Throughout the Lake Superior Basin, however, martens have a dynamic history of extirpation and reintroduction that has resulted in a complex configuration of local populations with unique genetic structures^37,38^. Indeed, following widespread local extirpations, martens have since been reintroduced to Wisconsin and Michigan from source populations in Minnesota and Ontario, respectively, while Wisconsin also received translocated martens from Colorado now known to be non-native Pacific martens (*Martes caurina*^39^) (Fig. 1a). Consequently, the management of Lake Superior martens, and Isle Royale in particular, is likely complicated by an amalgam of local and introduced lineages throughout the region.

Herein, we identified the source and colonization history of the recently rediscovered marten population on Isle Royale using population genetic and demographic analyses. We explored the phylogenetic history of martens on Isle Royale using nuclear and mitochondrial DNA from biological samples collected across the Lake Superior basin. Given the potential for endemism on Isle Royale and the complex genetic structure of marten populations regionally^37,38^, we then assessed the potential for Isle Royale martens as an evolutionarily significant unit (ESU^40,41^). Finally, we used demographic bottleneck tests to assess three putative colonization scenarios: (1) an historic, pre-settlement colonization; (2) a successful 1966 reintroduction; and (3) a modern colonization consistent with the timing of rediscovery. Due to their historic prevalence, and the ability of martens to maintain cryptic populations for millennia^19^, we hypothesized that the extant marten population on Isle Royale stemmed from a historical colonization and presents an endemic evolutionary lineage. We predicted significant structure among nuclear markers, with martens on Isle Royale representing a distinct genetic cluster, and we expected reciprocal monophyly across mtDNA sequences for martens on Isle Royale.

## Results

### Microsatellite analyses

We genotyped a total of 230 unique individuals across 6 sampled populations of martens (Table 1). Despite isolation, martens on Isle Royale exhibited no evidence of inbreeding; however, allelic richness was considerably lower than all other locations, heterozygosity was the second lowest of all populations, and only 1 unique allele was present. Alternatively, martens from Colorado (i.e., *M*. *caurina*) exhibited the highest proportion of unique alleles, while martens in Wisconsin revealed high degrees of both allelic richness and private alleles despite being a reintroduced, state endangered species^42^. All populations exhibited deviations from HWE, and Isle Royale and Wisconsin each exhibited linkage disequilibrium (Table S1, Supporting Information).

**Table 1:** Diversity of 14 microsatellite loci and 2 mtDNA genes used to characterize American martens (*Martes americana*) across 5 Lake Superior basin sites and Colorado (*Martes caurina*, introduced to northern Wisconsin).

Location	Microsatellite diversity						COI (174 bp)				CytB (370 bp)			
	*N*	*L*	*Ra*	*Pa* (%)	*H*_*Obs*_	*F*_*IS*_	*N*	*H*	*Hd*	*π*	*N*	*H*	*Hd*	*π*
Colorado	29	9	5.33	32.65	0.45	0.13	20	2	0.44	0.003	20	3	0.42	0.002
Isle Royale	27	9	3.67	3.03	0.52	−0.02	39	1	0.00	0.00	31	3	0.13	0.0004
Michigan	30	8	5.73	0.00	0.67	−0.01	20	1	0.00	0.00	20	3	0.35	0.001
Minnesota	64	14	5.53	10.99	0.56	0.13	20	1	0.00	0.00	20	4	0.44	0.002
Ontario	30	8	6.28	0.00	0.70	−0.01	20	2	0.10	0.0006	20	7	0.64	0.004
Wisconsin	50	14	6.26	18.81	0.55	0.17	17	2	0.49	0.003	18	2	0.29	0.003

Number of individuals genotyped (*N*), maximum number of loci genotyped (*L*), allelic richness (*Ra*), proportion of private alleles (*Pa*), observed heterozygosity (*H*_*Obs*_), and Fisher’s inbreeding coefficient (*F*_*IS*_) were calculated for microsatellites, while number of individuals sequenced (*N*), number of haplotypes (*H*), haplotype diversity (*Hd*), and nucleotide diversity (*π*) were calculated for the cytochrome *b* (COI) and cytochrome *c* oxidase subunit I (CytB) sequences.

### Population structure

**Figure 2:**
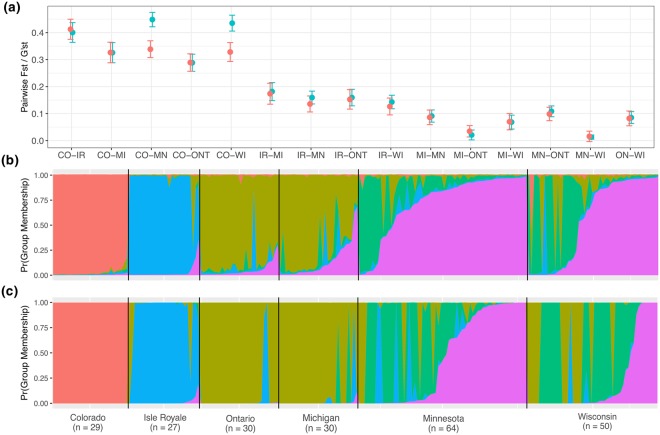
Population structure among microsatellites identified via pairwise *F*_*ST*_ (red) and *G*′_*ST*_ (blue) (**a**) Bayesian STRUCTURAMA analyses (**b**) and discriminant analysis of principle components (**c**). All methods identified Colorado (CO) and Isle Royale (IR) martens as distinct genetic clusters, while Michigan (MI), Minnesota (MN), Ontario (ONT), and Wisconsin (WI) exhibited more admixture.

Across sites, pairwise *F*_*ST*_ (0.02–0.40) and *G*′_*ST*_ (0.01–0.45) ranged widely, with martens from Isle Royale and Colorado displaying the largest differences (>0.1) from other populations (Fig. 2a). Reintroduced populations did not diverge substantially from their source populations, as FCA illustrated 3 distinct population clusters: Colorado, Minnesota-Wisconsin, and Isle Royale-Michigan-Ontario (Fig. 1b). However, FCA revealed fine-scale structure in the latter group with Isle Royale further segregating from Michigan and Ontario martens, suggesting that martens on Isle Royale diverged from the closest geographic population in Ontario (Fig. 1c). AMOVA similarly detected significant structure among sites and indicated fine-scale structure with Isle Royale as a 4^th^ distinct cluster (Table S2, Supporting Information).

Both the Bayesian and multivariate analyses of population structure identified 5 genetic clusters (Table S3, Supporting Information), and both classified martens from Isle Royale and Colorado as genetically unique populations (Fig. 2b,c). Conversely, the reintroduced populations in Michigan and Wisconsin exhibited considerable admixture with their respective source populations, Ontario and Minnesota, but no Lake Superior basin sites exhibited evidence of introduced alleles present in martens from Colorado (i.e., *M*. *caurina*). Structure analyses were consistent with and without the use of *M*. *caurina* as an outgroup (Fig. S1, Supporting Information).

### mtDNA analyses

**Figure 3:**
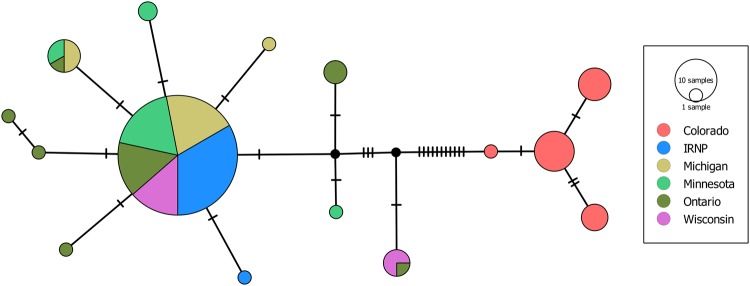
Median joining network for combined CytB and COI sequences. Tick marks indicate single nucleotide mutations. Colorado martens (*M*. *caurina*) were the only monophyletic group and Isle Royale (IRNP) was not distinct.

We successfully sequenced 137 individuals for the cytochrome *c* oxidase subunit I gene (COI) and 129 individuals for the cytochrome *b* gene (CytB). All sequenced scat samples were confirmed as martens via BLAST. All COI sequences included a section of 12 undetermined sites and were therefore concatenated to 174 bp fragments for all subsequent analyses. Haplotype and nucleotide diversity were low to non-existent for COI, as multiple locations exhibited a single haplotype (Table 1). Conversely, CytB exhibited moderate diversity with 25 segregating sites compared to 4 in COI. Minnesota and Ontario martens presented the most CytB haplotypes, however, this relationship was driven by a large number of singletons (Fig. 3). In total, there were no fixed polymorphisms for any Lake Superior martens, and only Colorado martens (i.e., *M*. *caurina*) exhibited reciprocal monophyly (Fig. 3). Moreover, the majority of martens in the Lake Superior basin, including Isle Royale, were represented by single haplotype for both COI (n = 110) and CytB (n = 83) (Fig. 3).

### Demographic analyses

Program MIGRAINE^43^ detected a significant bottleneck in martens on Isle Royale, with an observed *N*_*ratio*_ of 0.0007 and a 95% confidence interval (0.00027–0.11) that did not overlap 1. Further, MIGRAINE estimated an historic effective population size (*N*_0_) of 2947 (1208–14248) and a current effective population (*N*_1_) of 2.05 (2.00 − ∞), resulting in an estimated bottleneck time of 0.50 (0.0041–1.22) generations. Similarly, the loss of heterozygosity test from the source population in Ontario estimated that *N*_*e*_ = 1.94 for Isle Royale martens, while tests in the program LDNe^44^ estimated effective population sizes of 3.2 (2.2–7.6) and 257 (53 − ∞) for Isle Royale and Ontario, respectively.

## Discussion

Martens on Isle Royale exhibited considerable differentiation in nuclear markers from other regional marten populations, but mitochondrial sequences revealed no reciprocally monophyletic marten lineages within the Lake Superior Basin. Rather, martens from Isle Royale overwhelmingly shared mtDNA haplotypes with other populations. Thus, contrary to our hypothesis and despite significant population structure in nuclear markers across the region, we found no evidence of ESUs in martens of the Lake Superior Basin. This incongruity between nuclear and mitochondrial analyses indicates that while extant martens on Isle Royale are a distinct and isolated population, their colonization of the island was recent. Demographic analyses confirmed that martens on Isle Royale were subject to a recent population change and detected a significant bottleneck. Moreover, all demographic analyses showed that martens on Isle Royale are a small cohort (*N*_*e*_ ≅ 2) that derived from a much larger population of several hundred to thousands of individuals. This is consistent with our structure analyses showing that martens on Isle Royale were most closely related to individuals in Ontario, which is home to a large, panmictic marten population^45^. Thus, we conclude that the rediscovered marten population on Isle Royale stemmed from a recent but cryptic colonization via mainland Ontario. Any apparent genetic differentiation in nuclear markers is likely the result of founder effects, and the low allelic richness, limited number of unique alleles, and reduced heterozygosity of Isle Royale relative to Ontario support this conclusion. Given the estimated effective population size of approximately 2 individuals, contemporary Isle Royale martens were likely founded by a pair of colonizing individuals or a fertilized female.

Assuming the extant marten population on Isle Royale was founded contemporarily by way of Ontario, we tested three plausible colonization scenarios. Given the low mutation rates of CytB and COI in vertebrates^46,47^, it is possible that martens colonized Isle Royale within the last several hundred years and persisted undetected for much of the 20^th^ century^19^. Secondly, martens could have been reintroduced to Isle Royale from Ontario *c*. 1966, again going undetected for several decades^34^. Lastly, martens could have colonized Isle Royale in the late 20^th^ century via Ontario, the closest mainland (35 km), possibly using an ice bridge across Lake Superior, as has occurred for other Isle Royale carnivores^29,48^. Our MIGRAINE estimates indicate martens on Isle Royale experienced a significant bottleneck within the last generation (i.e. 5 years). While we know that extant martens have occupied Isle Royale for approximately 25 years^34^, samples used in this analysis were primarily collected from 2006–2009, putting the timing of colonization within a decade of marten rediscovery. This estimate is consistent with our other analyses that suggest martens colonized Isle Royale recently and are still recovering from a significant population bottleneck. Moreover, given the prevalence of ecological research on Isle Royale in the 20^th^ century^28,49^ it seems unlikely that martens would have gone undetected for decades following an earlier colonization event like the potential 1966 reintroduction. Thus, we postulate that martens were historically extirpated from Isle Royale but recolonized the island around the time of their rediscovery, *c*. 1991. Historical ice data shows numerous ice bridges connected Isle Royale to mainland Ontario in the 10 years preceding marten rediscovery^29^, thereby making a natural colonization possible. It is worth noting, however, that martens are the most widely translocated carnivore in North America^50^ and have a history of human-assisted island colonization – both sanctioned and unsanctioned^19^. Thus, human-assisted dispersal of martens to Isle Royale around the time of rediscovery cannot be precluded.

National Park Service policy aims to restore historical communities and ecosystem processes where appropriate, and the mammal community on Isle Royale appears to be an ideal candidate for reintroductions due to significant turnover in the last century^30^. For instance, wolves, coyotes (*Canis latrans*), and martens have all colonized the island, while Canada lynx, coyotes, and martens were also extirpated at one point or another^28^. The dominant herbivore, woodland caribou, was replaced by moose, and white-tailed deer (*Odocoileus virginianus*) were introduced but quickly extirpated. In addition, beavers (*Castor canadensis*) recolonized Isle Royale after apparent extirpation in the 19^th^ century^28,51^, and foxes were likely introduced for fur farming^28,52^. This dynamic history of colonization and extinction in an ecosystem that has been protected for most of the last century complicates the baseline for restoring historical Isle Royale communities and interactions. Moreover, such dynamics, coupled with the lack of historical and prehistorical information regarding past Isle Royale communities, has precipitated questions about the necessity of reintroducing or augmenting mammal populations on the island^26^. Our data suggests that martens, one of the smaller mammals on Isle Royale, were historically extirpated but recolonized the island. Dispersal ability in mammals is strongly correlated with body mass^53^, indicating that if martens did indeed immigrate to Isle Royale naturally, they were less likely to do so than other, larger-bodied carnivores. Moreover, ice bridges generally do not form until January^54,55^, months after the natal dispersal period in martens, and dispersal success is largely a function of available cover^56^, none of which is present over ice. Thus, if martens did naturally recolonize Isle Royale despite such barriers, the natural repatriation of larger carnivores may also be possible.

The frequency of ice bridges connecting Isle Royale to the mainland is decreasing due to climate change, thereby reducing the probability of natural immigration to the island for many species^29^. This loss of gene flow will ultimately reduce genetic diversity in Isle Royale mammal populations, the effects of which are already evident in wolves^57^. Consequently, population augmentation will likely be necessary to maintain genetic diversity in most extant Isle Royale community members (i.e., genetic rescue)^48^ and for any potentially reintroduced species^29^. It is therefore critical to understand the current genetic composition of other Isle Royale mammal populations in order to preserve potential endemic lineages or local adaptations^58,59^, and to identify sources for potential translocations. We conducted the most thorough evolutionary assessment of an historical Isle Royale mammal to date and found that extant martens do not constitute a unique genetic lineage and could ultimately be augmented from a number of marten populations in the Lake Superior Basin with which they share haplotypes, though Ontario was identified as the most closely related population. Regardless, martens on Isle Royale possess reasonably high heterozygosity and maintain low inbreeding coefficients despite a significant bottleneck, indicating that direct genetic management is currently unnecessary. Previous studies, however, found that other Isle Royale mammals like red squirrels and deer mice (*Peromyscus maniculatus*) also appear unique, but these divergences have only been described morphologically^36^ and via random amplifications of polymorphic DNA (RAPD)^60^, respectively. Our results show that population bottlenecks and founder effects can generate such putatively unique lineages, despite only recent divergence; thus, without a more complete assessment of evolutionary history, future management of these potentially endemic populations is uncertain. Furthermore, both red squirrels and deer mice, along with other Isle Royale mammals like snowshoe hares (*Lepus americanus*), have limited dispersal capabilities^61^, and more thorough genetic assessments of these populations are needed to assess colonization, historical community baselines, and the potential for human-assisted dispersal.

## Conclusions

Isle Royale National Park is a notable wilderness area with a pristine reputation and a storied history of ecological research. Yet, like many systems, Isle Royale has experienced significant anthropogenic change, and has a dynamic history of species colonization and extinction. Consequently, very little is known about the history of the island’s vertebrate community as a whole. Our study revealed an unexpected and dynamic pattern of extinction and recolonization for a small-bodied carnivore and illustrates that even federally protected or historically pristine ecosystems have experienced more community turnover than previously appreciated. Given that anthropogenic disturbances have driven the turnover of fauna globally, identifying the biogeographic origins of extant species and documenting historical community composition are critical guideposts to establishing restoration baselines^62^ and effectively managing both native and non-native species^18,19^. Our work also illustrates the importance of continued regional monitoring, the relevancy of historical surveys, and the need for genetic techniques to establish robust and defensible conservation targets. While often difficult to obtain, the combination of these approaches has proved particularly useful^19^, so we emphasize the importance of multiple, interdisciplinary stakeholders when establishing conservation and restoration programs. Finally, our findings show that martens, one of the least likely island colonizers, repatriated Isle Royale following extirpation, providing optimism for the natural recolonization of other extirpated or declining carnivore populations. Moreover, given that restoration initiatives often center around charismatic megafauna – species that generally possess the greatest dispersal power – conservationists are likely underrepresenting the natural colonization potential for most other taxa. Thus, future efforts aiming to re-establish island communities should first assess the colonization histories of smaller, more dispersal limited species, to inform and evaluate restoration efforts *a proiri*.

## Methods

### Sample collection and microsatellite analyses

To identify the source and colonization history of martens to Isle Royale, and to assess ESUs for the martens across the Lake Superior Basin, we analyzed biological samples from three distinct biogeographical regions (Fig. 1a). First, we collected scat samples on hiking trails across Isle Royale during summers from 2006–2008 and 2012–2013. Scats collected from 2006–2008 were stored in conical centrifuge tubes containing 95% ethanol and later dried, while scats from 2012–2013 were swabbed *a priori* using a cotton-tipped applicator to collect epithelial cells. We extracted DNA from all samples using commercially available kits (QIAGEN, Valencia, CA). Samples from 2006–2008 were identified as martens by Wildlife Genetics International (WGI; Nelson, BC, Canada) via sequencing of the 16S rRNA gene^63^, while samples from 2012–2013 were identified using fragment analysis of control region segments^64^. Second, we sampled populations of martens on the mainland surrounding Lake Superior to capture regional genetic diversity and potential sources to Isle Royale. Specifically, we used existing genotypes from hair, scat, and tissue samples to characterize the reintroduced marten population in the Chequamegon National Forest of Wisconsin and existing genotypes from tissue samples to characterize its source population in the Superior National Forest of Minnesota (Fig. 1a)^42^. We then used muscle tissue from trapper harvested martens to characterize the reintroduced marten population in the Upper Peninsula of Michigan, as well as its source population in Ontario (Fig. 1a). Lastly, we used Pacific marten (*Martes caurina*) muscle tissue from Colorado^37^ as an outgroup population to characterize the relative genetic diversity of martens in the Lake Superior basin and to assess the potential introduction of non-native alleles to the region. All sampling was approved by the University of Wisconsin Animal Care and Use Committee (A005239-A01) and conducted ethically under the guidelines established by the American Society of mammalogists^65^.

We used a set of 14 polymorphic microsatellite loci to genotype samples, including Ma1, Ma2, Ma5, Ma7, Ma8, Ma11, Ma14, Ma19, Gg3, Gg7, and Tt4^66^, as well as Mer022, Mer041, and Mvis072^67^. Polymerase chain reactions were conducted following Manlick *et al*.^42^, analyzed on an ABI 3730xl DNA analyzer (Applied Biosystems, Foster City, CA, U.S.A.), and scored using GeneMapper® (Applied Biosystems, V. 5.0). All samples were independently genotyped in duplicate to minimize potential genotyping errors^68^, and genotypes in disagreement were successively re-scored via independent PCRs until genotypes could be confirmed or the sample was consumed. All unresolved scores (i.e. mismatching) were censored at the locus in question, and genotypes were screened for potential allelic dropout and null alleles in program CERVUS^69^ throughout the scoring process. We calculated an overall genotyping error rate of 0.066. Genotypes were combined across studies^37,42^, therefore the number of loci analyzed varied by site (Table 1); however, sites limited to 8 or 9 loci contained the most polymorphic markers (Table S1, Supporting Information). Moreover, given the sensitivity of downstream analyses to missing data (e.g., multivariate analyses), all samples that failed to amplify at a minimum of 7 loci (50% genotyped) were discarded^70^.

Because all Isle Royale and some Wisconsin samples were collected noninvasively (see Manlick *et al*.)^42^, we identified unique individuals prior to population genetic analyses. We used all available samples to first generate a genotype accumulation curve in the R package *poppr*^71^ and, consistent with our genotyping procedure, determined that ≥7 markers were needed to identify unique individuals (Fig. S2, Supporting Information). We then performed an identity test across all samples using a maximum probability of identity threshold of 0.05 and the conservative estimator *P*_(*ID*)*sib*_ in program CERVUS^69,72^. All samples with *P*_(*ID*)*sib*_ > 0.05 were assumed to be the same individual and we consolidated them to a single multilocus genotype. Once samples were reduced to unique individuals, we tested all populations for deviations from Hardy-Weinberg Equilibrium (HWE) in *poppr* using permutation tests with 1000 iterations^71^, and we assessed linkage disequilibrium in Genepop^73^ using a sequential Bonferroni correction. Population metrics including observed and expected heterozygosity, allelic richness, private alleles, and inbreeding coefficients were calculated using the R packages *diveRsity*^74^ and *PopGenKit*^75^.

### Population structure

We quantified genetic structure between sampled populations by first calculating pairwise *F*_*ST*_ and the standardized metric G′_ST_^76^ with 95% confidence intervals using 1000 permutations in *diveRsity*^74^. We then performed a three-dimensional factorial correspondence analysis (FCA) in GENETIX v. 4.05^77^ to capture variation among individuals and we visually identified population clusters. Finally, we implemented a series of hierarchical AMOVAs^78^ in *poppr* to first test for significant population structure when considering each sampled population separately, and then to explore the amount of variance explained by combining clustered populations identified via FCA.

To quantitatively assign individuals to genetic clusters and estimate the number of unique marten populations (*K*) within the Lake Superior basin we employed Bayesian clustering models in STRUCTURAMA 2.0^79^. Unlike most clustering algorithms, STRUCTURAMA does not assume a fixed number of populations and instead makes *K* a random variable to estimate the number of populations under a given Dirichlet process prior, the mean expected number of populations *E*(*K*). We ran seven variations of this model with a prior *E*(*K*) ranging from 2 to 8 populations to test the sensitivity of model results to prior distributions. All models used a single MCMC chain with 10^6^ generations sampled every 1,000 steps and an additional 10% burn-in. Using the estimated *K* that maximized likelihood, we then employed the classic Pritchard *et al*.^80^ model with admixture and correlated allele frequencies for 10^5^ iterations with a 10% burn-in to assign individuals to genetic clusters.

Bayesian clustering models assume populations are in HWE and assign individuals to clusters that minimize disequilibrium; however, not all populations we analysed were in HWE (Table S1, Supporting Information)^42^. To account for this, we also employed a discriminant analysis of principle components (DAPC) in the r package *adegenet*^81,82^ to assign individuals to populations. DAPC is a multivariate statistical approach that does not assume HWE, but instead transforms genotypic data to principle components and assigns individuals to populations by maximizing variation between genetic clusters (see Jombart *et al*. 2010 for details)^82^. We calculated the number of principle components (*N* = 12) via alpha-score optimization, identified the number of populations using *K*-means clustering and Bayesian Information Criterion, and then used DAPC to generate assignment probabilities for all individuals^82,83^. Genetic structure diagrams were constructed for both the DAPC and STRUCTURAMA results and plotted using the R package *strataG*^84^.

### Mitochondrial sequence analyses

To identify potential evolutionarily significant units and further quantify genetic variation among marten populations in the Lake Superior Basin, we amplified and sequenced fragments of the cytochrome *b* (CytB) and cytochrome *c* oxidase subunit I (COI) mtDNA genes. We sequenced all unique Isle Royale individuals and 20 randomly chosen individuals from each mainland population. All mainland samples were restricted to high-quality tissue samples; however, individuals from Isle Royale were identified from scat samples that also included potential prey DNA, thereby precluding the use of generalized mtDNA primers previously used to characterize martens^85^. Consequently, we developed marten-specific primers and amplified a 370 bp fragment of CytB and a 186 bp fragment of COI (Methods S1, Table S4, Supporting Information). All PCR reactions used 3ul of template, 1uM of forward and reverse primers, 0.25 mM dNTPs, 200 μM 10x Qiagen PCR buffer, additional MgCl_2_ for total of 2.5 mM, 2 mg/mL of BSA, and 5 units taq/μL. PCR conditions used an initial denature of 95 °C for 3 min, followed by 40 cycles of 94 °C for 30 s, 51 °C for 20 s, and 72 °C for 45 s, and finished with a final elongation of 72 °C for 10 min. Fragments were sequenced in both the 5′ and 3′ directions on an ABI 3730xl capillary sequencer, chromatograms were visualized and cleaned using MEGA 7.0^86^, and sequences were aligned within MEGA 7.0 using the MUSCLE algorithm^87^. Sequences from martens sampled on Isle Royale (i.e., scat) were then entered into a GenBank nucleotide BLAST search to confirm species identity. All sequences were deposited in GenBank (accession nos. MH684021- MH684285).

We calculated haplotype and nucleotide diversity for CytB and COI in all sampled populations using the program POPART^88^. We then combined data for all samples successfully sequenced across both genes and assessed relatedness and monophyly among marten populations using a median-joining network developed in POPART.

### Historical demography

To estimate the timing of colonization we assessed the demographic history of Isle Royale martens by characterizing temporal changes in effective population size using the single population with variable size (OnePopVarSize) model in program MIGRAINE v. 0.5.2^43^. Migraine employs a class of importance sampling algorithms and a generalized stepwise-mutation model (GSM) for microsatellite loci to generate point estimates and 95% coverage confidence intervals for the scaled parameters ancestral population size (2*θ* = 2*N*_0_*μ*), current population size (2*θ* = 2*N*_1_*μ*), and time of the demographic change in generations (*T* = *T*/*2N*_1_). Using these parameters, ancestral (*N*_0_) and current (*N*_1_) effective population sizes were estimated assuming a marten microsatellite mutation rate (*μ* = 3 × 10^−4^)^19^ and then used to solve for *T*. We estimated an additional parameter, *N*_*ratio*_ (*N*_1_/*N*_0_), to quantify historical population expansion (>1) or contraction (<1), with significant demographic changes identified by estimates with 95% confidence intervals that did not overlap 1^43^. Because MIGRAINE is sensitive to the number of loci used^43^, we combined the aforementioned Isle Royale marten genotypes with additional loci independently genotyped by WGI for individual assignment for a previous study. Specifically, we used 27 individuals sampled on Isle Royale from 2006–2008 and combined 9 polymorphic loci used for structure analyses with 9 unique WGI loci for a total of 18 markers (Table S5, Supporting Information). All models were run using 3 replicates of 2000 points, with 3000 runs per point.

To further assess demographic variation of martens on Isle Royale we estimated the current effective population size (*N*_*e*_) based on the loss of heterozygosity^89^ and linkage disequilibrium^44^. Given that the timing of colonization is not known, we employed a simplified loss of heterozygosity model and estimated *N*_*e*_ following^89^

$${H}_{1}={H}_{0}(1-\frac{1}{2{N}_{e}})\,$$

where *H*_1_ was the observed heterozygosity of the identified source population and *H*_0_ was the observed heterozygosity of the Isle Royale population. Secondly, we used the linkage disequilibrium-based estimator of *N*_*e*_ in the program LDNe^44^ to estimate effective population sizes for the source and Isle Royale populations. This approach assumes discrete generations and therefore cannot estimate *N*_*e*_ directly; thus we interpreted LDNe results as an estimate of the breeding population size^90^. We applied a random mating model, used the conservative, unbiased threshold of 0.05 for lowest allele frequency (*P*_*crit*_)^44,91^, and calculated 95% CIs via permutation tests.

## Electronic supplementary material

Supporting Information

## Data Availability

All data are available through the Dryad Digital Repository (10.5061/dryad.m58q16m) and sequence data is available through GenBank (accession nos. MH684021–MH684285). All samples are housed in perpetuity at −80 °C at the University of Wisconsin-Madison.
