# Supplementary material for: Dynamic colonization history in a rediscovered Isle Royale carnivore
Source: Sci Rep. 2018 Aug 23;8:12711. doi: 10.1038/s41598-018-31130-0 (PMC6107671; doi:10.1038/s41598-018-31130-0)
Supplement: Supplementary file 1 — Supporting Information [file 41598_2018_31130_MOESM1_ESM.docx]

**Supplementary Information - Methods S1, Table S1-S5, Figures S1-S2**

**Dynamic colonization history in a rediscovered Isle Royale carnivore**

Philip J. Manlick^1^*, Mark C. Romanski^2^, Jonathan N. Pauli^1^

^1^ *University of Wisconsin – Madison, Department of Forest & Wildlife Ecology*

^2^ *National Park Service, Isle Royale National Park*

*Corresponding Author**:** pmanlick@wisc.edu, Department of Forest and Wildlife Ecology, University of Wisconsin – Madison, 1630 Linden Drive, Madison, WI 53706.

**Methods S1.**

To develop marten-specific primers we first accessed complete 1140bp CytB American marten (*Martes americana*) sequences from Stone et al. (2002)^1^ and 600-658bp COI marten fragments from the Barcode of Life initiative ^2^. To account for prey sequences potentially present in marten scats we also accessed complete 1140bp CytB sequences for 50 deer mice (*Peromyscus maniculatus*), 50 red squirrels (*Tamiasciurus hudsonicus*), 5 snowshoe hares (*Lepus americanus*), and 8 moose (*Alces alces)*, and we also used 600-658bp COI fragments from 20 deer mice, 20 red squirrels, 1 snowshoe hare, and 2 moose. All sequences were accessed via GenBank and aligned using the MUSCLE algorithm in MEGA7. For each gene, we used the program Primaclade ^3^ to identify conserved sequence pairs for marten primers. We then cross-checked all primer motifs with prey sequences and identified 2 primer pairs for each gene that minimized sequence overlap among martens and prey, particularly at the 3’ end. For all primers we assessed hairpins and self-dimers via OligoAnalyzer v. 3.1 (Integrated DNA Technologies), and then used Primer3 v. 0.4.0 ^4^ to confirm that primers matched known marten sequences. We then extracted DNA from 2 deer mice, 2 red squirrels, and 1 snowshoe hare collected by Carlson *et al.* (2014)^5^ and ran PCRs for all putative marten-specific primer pairs to test for cross amplification. PCR products were assessed via gel electrophoresis and primer pairs with the least non-specific amplification were selected for marten sequencing (Table S2).

**Table S1.** Summary of samples (N), number of alleles (k), polymorphic information content (PIC), observed heterozygosity (Ho), and expected heterozygosity (He) for all loci across sampled populations. Summary statistics in **bold** indicate loci that deviated from Hardy-Weinberg Equilibrium (α = 0.05) and statistics in *italics* indicate loci that exhibited linkage disequilibrium (α = 0.05; sequential Bonferroni correction).

|  | Summary (All) | | |  | Colorado | | |  | Isle Royale | | |  | Michigan | | |  | | Minnesota | | | |  | | Ontario | | | |  | | Wisconsin | | | |  |
| --- | --- | --- | --- | --- | --- | --- | --- | --- | --- | --- | --- | --- | --- | --- | --- | --- | --- | --- | --- | --- | --- | --- | --- | --- | --- | --- | --- | --- | --- | --- | --- | --- | --- | --- |
| Loci | N | k | PIC |  | k | Ho | He |  | k | Ho | He |  | k | Ho | He | |  | | k | Ho | He | |  | | k | Ho | He | |  | | k | Ho | He | |
| Gg3 | 229 | 10 | 0.62 |  | 2 | 0.07 | 0.07 |  | 2 | 0.29 | 0.25 |  | 6 | 0.50 | 0.54 | |  | | **8** | **0.56** | **0.65** | |  | | 6 | 0.77 | 0.71 | |  | | *8* | *0.60* | *0.59* | |
| Gg7 | 229 | 9 | 0.77 |  | **6** | **0.41** | **0.68** |  | 4 | 0.36 | 0.32 |  | 6 | 0.77 | 0.73 | |  | | 8 | 0.70 | 0.79 | |  | | 7 | 0.80 | 0.80 | |  | | *6* | *0.69* | *0.76* | |
| Ma1 | 127 | 21 | 0.87 |  | **10** | **0.74** | **0.79** |  | - | - | - |  | - | - | - | |  | | **10** | **0.70** | **0.83** | |  | | - | - | - | |  | | 14 | 0.81 | 0.86 | |
| Ma11 | 201 | 7 | 0.54 |  | - | - | - |  | 2 | 0.50 | 0.50 |  | 3 | 0.40 | 0.39 | |  | | 5 | 0.52 | 0.60 | |  | | 2 | 0.31 | 0.27 | |  | | ***3*** | ***0.29*** | ***0.55*** | |
| Ma14 | 222 | 11 | 0.75 |  | **6** | **0.54** | **0.71** |  | *4* | *0.63* | *0.75* |  | 7 | 0.86 | 0.74 | |  | | **7** | **0.57** | **0.67** | |  | | 6 | 0.60 | 0.69 | |  | | ***7*** | ***0.55*** | ***0.68*** | |
| Ma19 | 97 | 5 | 0.71 |  | - | - | - |  | - | - | - |  | - | - | - | |  | | 5 | 0.71 | 0.77 | |  | | - | - | - | |  | | *5* | *0.68* | *0.74* | |
| Ma2 | 225 | 8 | 0.75 |  | 7 | 0.69 | 0.79 |  | *4* | *0.69* | *0.70* |  | 5 | 0.83 | 0.79 | |  | | 6 | 0.68 | 0.73 | |  | | 6 | 0.76 | 0.79 | |  | | *7* | *0.71* | *0.73* | |
| Ma5 | 219 | 11 | 0.77 |  | 7 | 0.66 | 0.61 |  | 6 | 0.62 | 0.67 |  | 5 | 0.60 | 0.70 | |  | | **7** | **0.63** | **0.74** | |  | | **7** | **0.73** | **0.82** | |  | | 6 | 0.77 | 0.73 | |
| Ma7 | 132 | 7 | 0.38 |  | - | - | - |  | **3** | **0.33** | **0.35** |  | - | - | - | |  | | **4** | **0.36** | **0.41** | |  | | - | - | - | |  | | **6** | **0.28** | **0.43** | |
| Ma8 | 193 | 10 | 0.77 |  | - | - | - |  | *5* | *0.85* | *0.75* |  | **6** | **0.57** | **0.69** | |  | | **7** | **0.68** | **0.75** | |  | | 7 | 0.80 | 0.83 | |  | | 8 | 0.70 | 0.80 | |
| Mer022 | 94 | 12 | 0.54 |  | - | - | - |  | - | - | - |  | - | - | - | |  | | 5 | 0.53 | 0.52 | |  | | - | - | - | |  | | ***7*** | ***0.41*** | ***0.60*** | |
| Mer041 | 140 | 7 | 0.50 |  | 4 | 0.29 | 0.57 |  | - | - | - |  | - | - | - | |  | | 4 | 0.38 | 0.46 | |  | | - | - | - | |  | | ***6*** | ***0.50*** | ***0.55*** | |
| Mvis072 | 225 | 21 | 0.76 |  | 4 | 0.30 | 0.39 |  | 3 | 0.40 | 0.46 |  | 9 | 0.87 | 0.82 | |  | | 11 | 0.72 | 0.73 | |  | | 10 | 0.87 | 0.83 | |  | | ***13*** | ***0.65*** | ***0.78*** | |
| Tt4 | 143 | 8 | 0.40 |  | 3 | 0.35 | 0.30 |  | - | - | - |  | - | - | - | |  | | **4** | **0.05** | **0.11** | |  | | - | - | - | |  | | ***5*** | ***0.08*** | ***0.20*** | |
| Overall | 229 | 147 | 0.65 |  | 5.4 | 0.45 | 0.55 |  | 3.7 | 0.52 | 0.53 |  | 5.9 | 0.68 | 0.68 | |  | | 6.5 | 0.56 | 0.63 | |  | | 6.4 | 0.71 | 0.72 | |  | | 7.2 | 0.55 | 0.64 | |

**Table S2.** Analysis of molecular variance (AMOVA) results quantifying percentage of variation explained by population clusters (n) identified using factorial correspondence analysis. All results were significant (p<0.001).

| Covariance explained (%) | ^1^Independent (n=6) | ^2^FCA clusters (n=3) | ^3^Isle Royale (n=4) |
| --- | --- | --- | --- |
| Between sites | 9.00 | 9.06 | 10.47 |
| Within sites | 9.25 | 10.83 | 9.36 |
| Within Samples | 81.75 | 80.10 | 80.17 |

^1^ All sites independent

^2^ FCA clusters: Colorado, Isle Royale-Michigan-Ontario, and Minnesota-Wisconsin

^3^ FCA clusters with Isle Royale separate: Colorado, Isle Royale, Michigan-Ontario, Minnesota-Wisconsin

**Table S3.** Assignment probabilities and likelihood of genetic clusters derived from STRUCTURAMA analysis of 6 marten populations in the Lake Superior basin and Colorado. Models were assigned prior probabilities ranging from 2 expected clusters [E(K) = 2] to 8 expected clusters [E(K) = 2]. Bold numbers indicate highest probability for each model and model with maximum likelihood. All but one model estimated 5 genetic clusters.

| Clusters (K) | E(K) = 2 | E(K) = 3 | E(K) = 4 | E(K) = 5 | E(K) = 6 | E(K) = 7 | E(K) = 8 |
| --- | --- | --- | --- | --- | --- | --- | --- |
| 2 | 0.00 | 0.00 | 0.00 | 0.00 | 0.00 | 0.00 | 0.00 |
| 3 | 0.22 | 0.05 | 0.01 | 0.00 | 0.01 | 0.00 | 0.00 |
| 4 | **0.51** | 0.36 | 0.24 | 0.16 | 0.15 | 0.09 | 0.08 |
| 5 | 0.25 | **0.52** | **0.62** | **0.65** | **0.62** | **0.42** | **0.58** |
| 6 | 0.01 | 0.07 | 0.12 | 0.18 | 0.22 | 0.41 | 0.32 |
| 7 | 0.00 | 0.00 | 0.00 | 0.01 | 0.01 | 0.08 | 0.02 |
| 8 | 0.00 | 0.00 | 0.00 | 0.00 | 0.00 | 0.01 | 0.00 |
| Likelihood | -5997.9 | -5770.9 | -5469.3 | -5456.6 | -5750.9 | **-5441.0** | -5446.6 |

**Table S4.** Primers used to amplify CytB and COI fragments from marten scat.

| Primer | Sequence (5’ to 3’) |
| --- | --- |
| Cytb_370F | CTTTTGAGGTGCGACCGTA |
| CytB_370R | GCGGAATATCATGCCTCG |
| COI_186F | ATAATTGGGGGCTTCGGA |
| COI_186R | CACTGGCAGGGATAAGAGTAGAA |

**Table S5.** Summary of additional loci employed by WGI to genotype Isle Royale martens and used in MIGRAINE models.

| **^†^**Locus | *N* | Alleles | *H_O_* | *H_E_* | PIC |
| --- | --- | --- | --- | --- | --- |
| Ma2 | 33 | 4 | 0.67 | 0.74 | 0.68 |
| Ma7 | 33 | 2 | 0.36 | 0.34 | 0.28 |
| Ma9 | 33 | 1 | 0.00 | 0.00 | 0.00 |
| Ma10 | 33 | 3 | 0.64 | 0.68 | 0.59 |
| Ma18 | 33 | 5 | 0.67 | 0.64 | 0.56 |
| MP0055 | 33 | 3 | 0.55 | 0.58 | 0.49 |
| MP0059 | 33 | 3 | 0.64 | 0.64 | 0.55 |
| MP0085 | 33 | 3 | 0.64 | 0.51 | 0.39 |
| MP0114 | 33 | 1 | 0.00 | 0.00 | 0.00 |
| MP0175 | 13 | 1 | 0.00 | 0.00 | 0.00 |
| MP0197 | 33 | 4 | 0.73 | 0.73 | 0.67 |
| Mean | 33 | 2.72 | 0.44 | 0.44 | 0.38 |

**^†^**Locus = microsatellite loci; *N* = sample size; Alleles = number of alleles detected; *H_O_* = observed heterozygosity; *H_E_* = expected heterozygosity; PIC = polymorphic information content

**Fig S1.** Genetic structure in microsatellite loci among Lake Superior Basin marten populations (i.e. no *Martes caurina*) using factorial correspondence analysis (a) and the Bayesian clustering algorithm STRUCTURAMA (b).

**Figure S2.** Genotype accumulation curve denoting number of loci needed to identify individuals. All analyses used individuals with ≥ 7 loci genotyped which captures >95% of the total multilocus genotypes observed.

**References**

1. Stone, K. D., Flynn, R. W. & Cook, J. A. Post-glacial colonization of northwestern North America by the forest-associated American marten (Martes americana, Mammalia: Carnivora: Mustelidae). *Mol. Ecol.* **11,** 2049–63 (2002).

2. Adamowicz, S. J. International Barcode of Life: Evolution of a global research community. *Genome* **58,** 151–162 (2015).

3. Gadberry, M. D., Malcomber, S. T., Doust, A. N. & Kellogg, E. A. Primaclade - A flexible tool to find conserved PCR primers across multiple species. *Bioinformatics* **21,** 1263–1264 (2005).

4. Untergasser, A. *et al.* Primer3-new capabilities and interfaces. *Nucleic Acids Res.* **40,** 1–12 (2012).

5. Carlson, J. E. *et al.* Potential role of prey in the recovery of American martens to Wisconsin. *J. Wildl. Manage.* **78,** 1499–1504 (2014).
